# From Single Nutrients to Networked Health: Micronutrients and Bioactive Molecules in Development, Interaction, and Clinical Translation

**DOI:** 10.3390/nu18132201

**Published:** 2026-07-07

**Authors:** Jiaqiang Huang, Kongdi Zhu

**Affiliations:** Beijing Advanced Innovation Center for Food Nutrition and Human Health, Department of Nutrition and Health, China Agricultural University, Beijing 100083, China; zkd15938702692@163.com

Micronutrients, including essential minerals and vitamins, together with bioactive molecules such as polyphenols, flavonoids, and milk-derived proteins, are fundamental to human health. Their functions span enzymatic cofactors, redox regulators, signaling modulators, and epigenetic influencers. Despite decades of research, two major challenges persist: translating mechanistic insights into safe and effective nutritional products, and moving beyond single-nutrient thinking toward an integrated understanding of nutrient–nutrient and nutrient–host interactions. This Special Issue, “Micronutrients and Bioactive Molecules: Their Development, Interaction, and Impact on Human Health,” brings together a collection of original articles and reviews that address these challenges from complementary perspectives. These include developmental selenoprotein programming, trace element networks, and flavonoid-based cancer therapy. Collectively, these contributions underscore a paradigm shift toward precision, synergy, and systemic integration.

A robust understanding of the relationship between micronutrient status and disease outcomes requires large-scale population-based studies. The essential role of selenium in human health has been extensively reviewed [1], providing a broad context for the selenoprotein-focused studies in this issue.

The mechanistic foundation of micronutrient action is perhaps best exemplified by the selenoprotein family. Wang et al. [2] systematically quantified the mRNA expression of all 24 murine selenoprotein genes across embryonic and postnatal stages in the heart, brain, liver, and kidney. They identified three key patterns: deiodinases and thioredoxin reductases show limited embryonic expression, whereas glutathione peroxidases and biosynthesis-related genes undergo massive postnatal upregulation (up to 600-fold). These spatiotemporal data provide a roadmap for understanding why selenium deficiency during specific developmental windows leads to irreversible damage. The selenoprotein family, as comprehensively reviewed elsewhere [3], serves as a paradigm for understanding how trace elements exert pleiotropic effects through genetically encoded proteins. Extending these molecular insights to clinical applications, Liu et al. [4] critically reviewed supranutritional selenium supplementation in metabolic dysfunction-associated steatotic liver disease (MASLD). They concluded that while selenium holds promise, its narrow therapeutic window—deficiency on one side and insulin resistance or selenosis on the other—necessitates accurate baseline status assessment and the use of safer forms such as organic selenium or selenium nanoparticles.

Beyond individual elements, trace elements operate as interconnected networks. Pavić Vulinović et al. [5] offered an integrative overview of the zinc functional interactome across the hallmarks of health, emphasizing how zinc’s structural, catalytic, and regulatory roles intersect with other minerals and vitamins. The immunomodulatory functions of zinc, extensively discussed elsewhere [6], further support a network pharmacology approach. They argue that the fragmented literature on zinc can be consolidated using artificial intelligence-based multi-scale analyses, a forward-looking perspective that resonates throughout this Special Issue.

Bioactive molecules derived from milk, plants, or microbial fermentation often exert effects that exceed the sum of their individual components. Wang et al. [7] investigated silybin, a flavonoid derived from milk thistle, in a mouse model of post-myocardial infarction heart failure. They found that silybin ameliorated cardiac dysfunction and remodeling by suppressing HIF-1α-driven glycolysis, reducing lactate production, and restoring ATP levels. This finding adds to the growing evidence that flavonoids can reprogram energy metabolism in stressed hearts.

Polyphenols remain a cornerstone of bioactive molecule research. Wang et al. [8] performed an integrated bibliometric and mechanistic scoping review covering 12 polyphenol classes and seven major neurodegenerative diseases. Using keyword co-occurrence, clustering, and BERTopic modeling, they identified four persistent research hotspots: antioxidant and neuroprotective effects, anti-inflammatory signaling, cognition and aging with sex-specific responses, and animal models of memory impairment. The conceptual framework of polyphenols as more than direct antioxidants, proposed elsewhere [9], aligns with the multi-target mechanisms identified by these authors. Moreover, the metabolic fate of polyphenols in the gut is strongly influenced by the intestinal microbiota, which determines their bioavailability and biological activity [10]. They concluded that, despite the multi-target neuroprotective potential of polyphenols, limited bioavailability and heterogeneous trial designs remain major barriers, calling for advances in delivery systems and precision nutrition.

Delivery systems themselves are emerging as a critical determinant of therapeutic efficacy. Food-derived exosomes (FEVs), natural nanovesicles from plants and milk, have recently been recognized as promising oral platforms for colon-targeted delivery [11]. Unlike synthetic nanoparticles, FEVs exhibit intrinsic stability against gastric acid and digestive enzymes, low immunogenicity, and excellent biocompatibility. Their lipid bilayer membranes, rich in phosphatidic acid and sphingolipids, enable efficient transmembrane transport via receptor-mediated endocytosis or direct membrane fusion. Engineered FEVs incorporating polysaccharide coatings, reactive oxygen species-responsive surface modifications, or hydrogel encapsulation can deliver polyphenols, nucleic acids, or small-molecule drugs precisely to inflamed colonic tissues in inflammatory bowel disease and colorectal cancer. This paradigm of using nature-derived nanocarriers for precision nutrient delivery bridges food science, nanotechnology, and clinical nutrition, offering a roadmap for translating bioactive molecules into next-generation therapeutic products.

Dietary substances do not act in isolation; they signal through the enteric nervous system (ENS), often referred to as the “second brain.” Wang et al. [12] provided a systematic review of how polyphenols, short-chain fatty acids, amino acids, prebiotics, and probiotics modulate ENS functions, including gastrointestinal motility, neuronal survival, neuro-immune homeostasis, and the gut–brain axis. They highlighted current limitations, such as reliance on rodent models and the unclear mechanisms of the human ENS, and proposed future directions involving single-cell sequencing, targeted delivery systems, and precision medicine. This review serves as a conceptual bridge, linking the molecular actions of micronutrients and bioactives to systemic physiological regulation.

The gut microbiota is not merely a passive recipient of dietary nutrients; it actively shapes micronutrient bioavailability and, in turn, is shaped by them. Iron exemplifies this bidirectional crosstalk [13]. Hosts employ a strategy known as “nutritional immunity”, using hepcidin, calprotectin, lipocalin-2, and lactoferrin to sequester iron from invading pathogens, whereas commensal bacteria produce siderophores to acquire this essential metal. More strikingly, microbial metabolites directly regulate host iron absorption. For example, short-chain fatty acids lower luminal pH to enhance iron solubility, while metabolites such as 1,3-diaminopropane and reuterin suppress HIF-2α, the master transcriptional regulator of intestinal iron uptake. Iron supplementation, while correcting deficiency, may inadvertently promote the expansion of pathobionts such as *Escherichia coli* and *Clostridium difficile*. Conversely, iron deficiency reduces beneficial butyrate-producing Firmicutes. This iron–microbiome–host triad illustrates a general principle: micronutrient interventions must consider the pre-existing microbial ecosystem, which may dictate therapeutic outcomes. These insights call for microbiome-informed personalized nutrition strategies, an approach directly applicable to selenium, zinc, and other trace elements discussed throughout this Special Issue.

The contributions in this Special Issue collectively advance micronutrient and bioactive molecule research along three axes: first, from association to mechanism, as illustrated by selenoprotein developmental mapping; second, from single agents to synergistic systems, as seen in flavonoid-nanocarrier platforms and food-derived exosome-based delivery; and third, from population averages to individual responsiveness, highlighted by the narrow therapeutic window of selenium. Priority areas for future research include the development of validated multi-marker panels for assessing nutritional status, the integration of organoid and gut-on-a-chip models for mechanistic validation, and the application of artificial intelligence to map nutrient interaction networks across health and disease. Furthermore, the translation of bioactive molecules from bench to bedside must account for food processing: thermal treatments, enzymatic oxidation, and interactions with macronutrients can dramatically alter polyphenol profiles and bioactivities [14,15]. The rigorous characterization of processed food matrices is therefore essential for interpreting clinical outcomes. Finally, as compellingly argued elsewhere [16], the field of nutrition research must transition from empirical observations to hypothesis-driven, mechanistic science—requiring standardized nomenclature, the rigorous chemical characterization of experimental diets, and multivariate analyses to control for confounders. Only such methodological rigor will elevate nutritional science to the level of pharmacology, enabling the identification of specific nutrition-relevant molecules with drug-like health effects [17,18,19]. We hope this Special Issue will catalyze interdisciplinary collaboration, ultimately translating the science of micronutrients and bioactive molecules into safer, more effective, and more personalized strategies for human health.

We sincerely thank all authors for their outstanding contributions, the reviewers for their rigorous evaluations, and the editorial team of *Nutrients* for their unwavering support.
